# Trophic Relationships between the Parasitic Plant Species *Phelipanche ramosa* (L.) and Different Hosts Depending on Host Phenological Stage and Host Growth Rate

**DOI:** 10.3389/fpls.2016.01033

**Published:** 2016-07-13

**Authors:** Delphine Moreau, Stéphanie Gibot-Leclerc, Annette Girardin, Olivia Pointurier, Carole Reibel, Florence Strbik, Mónica Fernández-Aparicio, Nathalie Colbach

**Affiliations:** Agroécologie, AgroSup Dijon, INRA, Université de Bourgogne Franche-ComtéDijon, France

**Keywords:** *Phelipanche ramosa*, *Brassica napus*, *Geranium dissectum*, *Capsella bursa-pastoris*, weed, biomass, host, parasite

## Abstract

*Phelipanche ramosa* (L.) Pomel (branched broomrape) is a holoparasitic plant that reproduces on crops and also on weeds, which contributes to increase the parasite seed bank in fields. This parasite extracts all its nutrients at the host’s expense so that host–parasite trophic relationships are crucial to determine host and parasite growth. This study quantified the intensity with which *P. ramosa* draws assimilates from its host and analyzed whether it varied with host species, host phenological stage and host growth rate. A greenhouse experiment was conducted on three host species: the crop species *Brassica napus* (L.) (oilseed rape) and two weed species, *Capsella bursa-pastoris* (L.) Medik. and *Geranium dissectum* (L.). Plants were grown with or without *P. ramosa* and under three light levels to modulate host growth rate. The proportion of host biomass loss due to parasitism by *P. ramosa* differed between host species (at host fructification, biomass loss ranged from 34 to 84%). *B. napus* and *C. bursa-pastoris* displayed a similar response to *P. ramosa*, probably because they belong to the same botanical family. The sensitivity to *P. ramosa* in each host species could be related to the precocity of *P. ramosa* development on them. Host compartments could be ranked as a function of their sensitivity to parasitism, with the reproductive compartment being the most severely affected, followed by stems and roots. The proportion of biomass allocated to leaves was not reduced by parasitism. The proportion of pathosystem biomass allocated to the parasite depended on host species. It generally increased with host stage progression but was constant across light induced-host growth rate, showing that *P. ramosa* adapts its growth to host biomass production. The rank order of host species in terms of sink strength differed from that in terms of host sensitivity. Finally, for *B. napus*, the biomass of individual parasite shoots decreased with increasing their number per host plant, regardless of host growth rate. Results will be incorporated into a mechanistic model in order to analyze the effect of parasitic plant species on weed community assembly and to design new cropping systems for controlling *P. ramosa*.

## Introduction

Broomrapes are root-parasitic plant species of the *Phelipanche* and *Orobanche* genera that can cause severe yield losses to economically important crop species all over the world (Dhanapal et al., 1996; Parker, 2009). In France, branched broomrape – *Phelipanche ramosa* (L.) Pomel (syn. *Orobanche* *ramosa*) – is a frequent and harmful parasitic plant species with a large range of hosts including Solanaceae, Brassicaceae, and Fabaceae species. Among arable crops, *Brassica napus* (L.) (oilseed rape) is the favorite *P. ramosa* host with yield losses up to 90% (Gibot-Leclerc et al., 2012). *P. ramosa* reproduces not only on crop species but also on weed species (Boulet et al., 2001). Thus, even in the absence of a host crop, weeds can allow an increase in *P. ramosa* soil seed bank which can then infest a subsequent crop. To date, no efficient method is available for controlling broomrape in arable crops and integrated management practices are required (Dhanapal et al., 1996).

*P. ramosa* is a root-holoparasitic (i.e., chlorophyll-lacking) angiosperm that connects with the vascular system of its host’s roots and extracts all its nutrients (carbohydrates and minerals) and water at the host’s expense (Parker and Riches, 1993). Consequently, host–parasite trophic relationships are crucial to determine the growth and seed production of the two interacting plants and, therefore, the harmfulness of *P. ramosa*. For holoparasitic plants, host–parasite trophic relationships have never been compared for different host species under the same experimental conditions. Several studies dealing with trophic relationships in single broomrape–host combination pairs revealed that the deleterious effect of broomrape parasitism on host biomass production and distribution among host compartments (leaves, stems, and roots) varies across host and parasitic species, number of parasites and growing conditions (Barker et al., 1996; Dale and Press, 1998; Hibberd et al., 1998; Manschadi et al., 2001; Lins et al., 2007; Mauromicale et al., 2008). In the particular case of *P. ramosa*, host–parasite trophic relationships were studied for only one host species, *Solanum lycopersicum* L. (tomato) (Mauromicale et al., 2008). The trophic relationships have never been characterized for this parasite with either *B. napus* (*P. ramosa*’s favorite arable host crop in France) or weed species. Yet, such knowledge is crucial to better understand the impact of *P. ramosa* on *B. napus* production, both directly (by nutrient withdrawal from *B. napus* plants) and indirectly (via weed parasitism increasing *P. ramosa* soil seed bank) and thereby to parametrize simulation models allowing to identify cropping systems adapted to the control of *P. ramosa* (Colbach et al., 2011, 2014).

The trophic relationships can be characterized by, on the one hand, the response of the host plant to parasitism, referring to both number of parasite attachments and host growth reduction due to parasitism (Mauromicale et al., 2008) and, on the other hand, by the parasite sink strength, i.e., the proportion of the parasite biomass or growth rate relative to that of the host plant or the pathosystem (Lins et al., 2007; Hautier et al., 2010). Previous studies on host–parasite trophic relationships comparing different host species concerned hemiparasitic plant species (Matthies, 1996; Cameron et al., 2006; Hautier et al., 2010). In contrast to holoparasitic plant species like *P. ramosa*, hemiparasitic plant species are photosynthetically active, relying on the host for mineral nutrients, water and sometimes for carbohydrates (Watling and Press, 2001; Irving and Cameron, 2009). For these parasites, host growth reduction due to parasitism was shown to depend on host species (Cameron et al., 2006) while the analysis of the parasite sink strength showed that the parasite growth rate, and hence parasite biomass, increased with host growth rate (Hautier et al., 2010). Host species parasitized by a hemiparasitic plant were shown to be ranked the same, both in terms of host response to parasitism and parasite sink strength (Matthies, 1996).

Focusing on a holoparasitic plant species, the objective of the present study was to quantify the intensity with which *P. ramosa* draws assimilates from *B. napus*, its favorite arable host crop in France, and to analyze whether this intensity varies with host phenological stages and light intensity-induced host-growth rate. Our objective was also to compare the responses and effects of *P. ramosa* in two additional host species with differing levels of ability to promote parasite growth. The following research hypotheses were tested. Firstly, for a given host species, we assumed a constant response to parasitism, meaning that the growth of parasitized host plants will be proportional to the growth of healthy host plants, whatever the host stage, the host growth rate and the number of attached parasites. Secondly, for a given host species, we assumed a constant sink strength of the parasite, meaning that the growth of *P. ramosa* will be proportional to that of the pathosystem including host and parasite, irrespective of host stage, host growth rate and number of parasite attachments. Plant biomass will be considered as a proxy of assimilate fluxes to analyze host–parasite trophic relationships. These hypotheses were tested on host species grown in the presence or absence of *P. ramosa* in order to evaluate the impact of parasitism on host growth and under three light levels in order to generate different host growth rates. Oilseed rape (*B. napus*) was used as the preferred host crop species that supports numerous attachments and aboveground shoots of *P. ramosa* (Gibot-Leclerc et al., 2012). *Capsella bursa-pastoris* (L.) and *Geranium dissectum* (L.) were also used. They are both host weed species that support a limited number of viable attachments of *P. ramosa* (Boulet et al., 2001). Attachments generally result in aboveground parasite shoots on *C. bursa-pastoris* (Boulet et al., 2001) but more erratically on *G. dissectum*: no emergence was observed in greenhouse conditions (Boulet et al., 2001), while emerged shoots were reported in the field (Supplementary Data Sheet 1). Host reponse to parasitism was analyzed in terms of *P. ramosa* attachments, total host biomass production and biomass partitioning among host compartments (i.e., leaves, stems, roots, and reproductive compartments). The sink strength of the parasite was analyzed in terms of *P. ramosa* biomass production relative to that of the pathosystem as well as parasite shoot number per host plant and average parasite shoot biomass.

## Materials and Methods

### Experimental Treatments

A greenhouse experiment was conducted in Dijon (France) from September 2013 to July 2014 with three host species, two parasite seed densities, three light conditions and three replicates at each harvest date. The three host species were *B. napus* (Aviso cultivar, seeds from SW Seeds), *C. bursa-pastoris* and *G. dissectum* (seeds from Herbiseed). There exist several pathovar for *P. ramosa* (Le Corre et al., 2014). Here, the pathovar specific to the most cultivated French arable crop species (i.e., oilseed rape) was chosen. Parasite seeds were collected in 2002 from severely infested oilseed rape fields and identified as belonging to the *B. napus* pathovar (Le Corre et al., 2014). Plants of the three host species were grown without and with *P. ramosa* seeds (165 mg of *P. ramosa* seeds per pot, corresponding to ca. 55000–60000 seeds). Hereafter, plants of both treatments are referred as healthy and parasitized plants, respectively. Plants were also grown under three light levels (100, 34, and 29% daylight). The 100% light level corresponded to natural light whereas the 34 and 29% light levels were obtained using two types of shading net allowing to artificially reduce light levels over the plots^1^ The experimental design was a complete factorial design with three replicates. For each of the 18 experimental treatments (3 host species × 2 *P. ramosa* seed densities × 3 light levels), 12 plants were grown (4 harvest dates × 3 replicates), resulting in a total of 216 plants in the experiment.

### Cultural Conditions

Two L-pots were filled with 0.4 L of clay balls at the bottom and 1.6 L of a substrate made up of 1:3 of Biot sand and 2:3 of a disinfected soil (collected at Experimental station of Dijon-Epoisses, France) determined as 8% sand, 57% silt, 35% clay. For the treatment with *P. ramosa*, parasite seeds were mixed with all of the substrate moistened beforehand. Host plant seeds were sown 14 days later to allow preconditioning of *P. ramosa* seeds, i.e., the exposure of parasite seeds to temperate and moist conditions to make them more susceptible to host root exudates (Musselman, 1980). For each host species, one host seed was sown per pot on September 12th 2013. As *G. dissectum* seedlings did not emerge and grow correctly, a new batch of seeds was sown 4 months later. *G. dissectum* plants were grown during 6 weeks in a growth chamber to mimic greenhouse conditions similar to those experienced by the two other species, before transfer into the greenhouse in March.

Tap water was provided at frequencies and quantities that were adjusted to maintain humidity at about 70% of soil water-holding capacity. The water loss through evapotranspiration was estimated every 1–3 days by weighing reference pots (one per experimental treatment). Whenever the water content of the soil was below 70% of soil water-holding capacity, the pots were irrigated. In addition, 10 mL of a nutrient solution (10-10-10 corresponding to N-P-K) was provided once a week per pot. Our objective was to obtain environmental conditions as close as possible to those in the field. Thus, neither heating nor artificial light were used. Air temperature (PT100 sensors; Pyro-Contrôle, Vaulx-en-Velin, France) and incident photosynthetically active radiation (PAR; silicium sensors; Solems, Palaiseau, France) measurements were taken every 600 s and stored in a data logger (DL2e; Delta-T Devices, Cambridge, England). For *B. napus* and *C. bursa-pastoris*, the mean daily temperature (including night temperatures) was 13.3°C (ranging from 3.3 to 28.7°C), the mean daily incident photosynthetic active radiation for the 100% light level treatment was 8 mol m^-2^ day^-1^ (ranging from 1 to 33 mol m^-2^ day^-1^) and day length was 12.0 h (ranging from 8.2 to 16.1 h). For *G. dissectum* during the whole growth period (including in growth chamber), the mean daily temperature was 16.7°C (ranging from 6.2 to 28.7°C), the mean daily incident photosynthetic active radiation for the 100% light level treatment was 8 mol m^-2^ day^-1^ (ranging from 2 to 11 mol m^-2^ day^-1^) and day length was 14.3 h (ranging from 11.2 to 16.1 h).

### Plant Measurements

Plants were harvested at four phenological stages of the host species: rosette, elongation, flowering, and fructification. As parasitism did not delay host phenology, parasitized and healthy plants were harvested at the same date for a given host species in a given light treatment (Supplementary Data Sheet 2). Biomass was determined after drying for 48 h at 80°C with a balance precision up to 10^-5^ g. Host biomass was measured separately for roots, stems, leaves, and reproductive organs (including flowers and fruits). For *P. ramosa*, the number of attached individual parasitic plants and the number of aboveground *P. ramosa* shoots per host plant were counted and aboveground and belowground biomass per host plant were determined separately.

### Statistical Analyses

Analyzed variables were: total host biomass, host biomass per compartment (i.e., roots, stems, leaves, and reproductive organs), aboveground and belowground parasite biomass per host plant and number of aboveground shoots of *P. ramosa* per host plant. Analysis of variance was performed to study the effects of experimental factors, i.e., host species, *P. ramosa* parasitism, light level and host phenological stage, and their interactions on growth variables. Analysis of covariance was performed to analyze correlations between growth variables in relation to experimental factors. Significance was determined using α = 0.05 and analyses were performed using the lm function of R x64 3.0.3 (R Development Core Team, 2014).

## Results

### Parasite Number and Phenology Across Host Species

The number of attached *P. ramosa* plants per host plant differed between host species (*P* < 0.001), light levels (*P* < 0.001) and host phenological stages (*P* = 0.002; **Table 1**). *P. ramosa* infection success was the highest in *G. dissectum*, especially until flowering, followed by *B. napus* while it was low for *C. bursa-pastoris*. For *B. napus* and *G. dissectum*, the number of attachments decreased with decreasing light level (*P* < 0.003) and, for *G. dissectum* only, it varied with host phenological stage, being maximal at elongation (*P* < 0.001).

**Table 1:** Number of attached parasites for the three host species, the four phenological stages and the three light levels.

Host species	Light level	Rosette	Elongation	Flowering	Fructification
*Brassica napus*	100%	27.7	25.0	8.7	35.3
	34%	10.7	10.7	17.7	17.7
	29%	0.0	4.3	0.7	4.7
*Capsella bursa-pastoris*	100%	0.3	0.3	1.3	2.0
	34%	1.0	1.0	0.7	3.3
	29%	0.0	0.3	0.0	0.3
*Geranium dissectum*	100%	47.0	108.7	21.7	30.7
	34%	20.0	34.3	7.7	6.3
	29%	14.0	19.7	14.7	3.3

For the three host species, *P. ramosa* aboveground shoot number per host plant was maximal at the host fructification stage. It was much lower for *G. dissectum* (0.4 parasite shoots per host plant) and *C. bursa-pastoris* (1) than for *B. napus* (11) averaged over all light levels. So, despite the high number of *P. ramosa* attachments on *G. dissectum*, the success of *P. ramosa* to develop on this host species was low. Synchronization between host and parasite varied among species, with *P. ramosa* development, expressed relatively to host development, being later on *C. bursa-pastoris* (**Table 2**). Whatever the host species, the parasite did not have the time to fructify during the experiment.

**Table 2:** Synchronization between host and parasite phenology.

Host species	Light level	Host stage at parasite emergence	Host stage at parasite flowering
*Brassica napus*	100%	Flowering	Fructification
	34%	Flowering	Fructification
	29%	Flowering	Fructification
*Capsella bursa-pastoris*	100%	Fructification	-	
	34%	Fructification	Fructification
	29%	-	-
*Geranium dissectum*	100%	Fructification	-	
	34%	Flowering	Flowering
	29%	Flowering	Flowering

### Pathosystem Biomass Production

**FIGURE 1:**
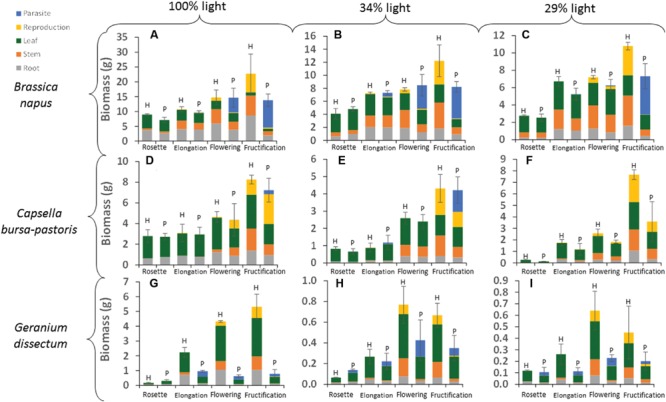
**Absolute values of biomass per compartment for each host species × host phenological stage × light level combination.** H is for healthy plants and P for parasitized plants. Data are means for three independent replicates. SE is represented for pathosystem biomass values (i.e., the combined host and parasite biomass in parasitized plants, or the sole host biomass in healthy plants). The part-labels from **(A)** to **(I)** show the different host species per light level combinations.

Host species, light level and host phenological stage all affected total pathosystem biomass production, i.e., the combined host and parasite biomass in parasitized plants, or the sole host biomass in healthy plants (*P* < 0.001). In addition, parasitism significantly decreased the pathosystem biomass (*P* < 0.001; **Figure 1**). This pathosystem biomass loss did not differ among host species (*P* = 0.53) but it varied with host phenological stage (*P* < 0.001). For *B. napus* and *C. bursa-pastoris* from rosette to flowering, pathosystem biomass was not or only little affected by parasitism. The effect was either non-significant or associated to a very low partial *R*^2^ (**Table 3**) while parasitism had a significant effect on pathosystem biomass at fructification stage **Table 3** At host fructification, pathosystem biomass loss was 36 and 26% for *B. napus* and *C. bursa-pastoris*, respectively (average over all light levels). For *G. dissectum*, parasitism decreased pathosystem biomass at all host phenological stages (**Table 3**), with the largest effect at host flowering and fructification stages where pathosystem biomass was respectively reduced by 78 and 79% compared to healthy host plants (average over all light levels). For the three species, the deleterious effect of parasitism on pathosystem biomass production also varied with light level (*P* < 0.001; **Figure 1**). It increased with light level for both *B. napus* and *G. dissectum*, while it was the strongest at the lowest light level for *C. bursa-pastoris*.

**Table 3:** Effects of parasitism by *Phelipanche ramosa*, light level and interaction on pathosystem biomass for each host species and phenological stage.

Host species	Factors	Rosette	Elongation	Flowering	*Fructification*
*Brassica napus*	Light level	0.89^∗∗∗^	0.85^∗∗∗^	0.97^∗∗∗^	0.48^∗∗^
	Parasitism	ns	0.04^∗^	ns	0.21^∗∗^
	Interaction	0.05^∗^	ns	ns	ns
*Capsella bursa-pastoris*	Light	0.93^∗∗∗^	0.69^∗∗∗^	0.64^∗∗^	0.45^∗∗^
	Parasitism	ns	ns	ns	0.17^∗^
	Interaction	ns	ns	ns	0.16^∗^
*Geranium dissectum*	Light level	0.47^∗∗^	0.73^∗∗∗^	0.42^∗∗∗^	0.45^∗∗∗^
	Parasitism	0.15^∗^	0.10^∗∗∗^	0.27^∗∗∗^	0.21^∗∗∗^
	Interaction	ns	0.13^∗∗∗^	0.30^∗∗∗^	0.29^∗∗∗^

*Partial *R*^2^-values determined by analysis of variance. ns for a non-significant effect, ^∗^*P* < 0.05; ^∗∗^*P* < 0.01; ^∗∗∗^*P* < 0.001.*

### Host Biomass Production

Host species, light level, host phenological stage and parasitism by *P. ramosa* all affected total host biomass (*P* < 0.001; **Figure 1**). In addition, the effect of parasitism differed among host species (*P* < 0.001) and host phenological stages (*P* < 0.001). For *B. napus* and *G. dissectum*, parasitism affected host biomass at all phenological stages except rosette stage while for *C. bursa-pastoris* it affected host biomass at fructification stage only (**Table 4**). At fructification, host biomass was reduced by ca. 84% for *G. dissectum*, 76% for *B. napus* and 34% for *C. bursa-pastoris* (average over all light levels). Finally, the deleterious effect of parasitism increased with light availability for both *B. napus* and *G. dissectum*, whereas it was strongest at the intermediate light level for *C. bursa-pastoris* (*P* < 0.001). For *B. napus*, the effect of the parasitism increased with host phenological stage while that of the light level diminished. This is shown by the increase of partial *R*^2^-values related to parasitism and the concomitant decrease of the partial *R*^2^-values related to the light level with progression of phenological stages (**Table 4**).

**Table 4:** Effects of parasitism by *Phelipanche ramosa*, light level and interaction on total host biomass for each host species and phenological stage.

Host species	Factors	Rosette	Elongation	Flowering	*Fructification*
*Brassica napus*	Light level	0.89^∗∗∗^	0.83^∗∗∗^	0.61^∗∗∗^	0.16^∗^
	Parasitism	ns	0.07^∗∗^	0.19^∗∗^	0.60^∗∗∗^
	Interaction	ns	ns	ns	ns
*Capsella bursa-pastoris*	Light level	0.93^∗∗∗^	0.70^∗∗∗^	0.64^∗∗^	0.50^∗∗∗^
	Parasitism	ns	ns	ns	0.26^∗∗∗^
	Interaction	ns	ns	ns	ns
*Geranium dissectum*	Light level	0.55^∗∗∗^	0.56^∗∗∗^	0.37^∗∗∗^	0.42^∗∗∗^
	Parasitism	ns	0.18^∗∗∗^	0.31^∗∗∗^	0.23^∗∗∗^
	Interaction	ns	0.22^∗∗∗^	0.31^∗∗∗^	0.31^∗∗∗^

*Partial *R*^2^-values determined by analysis of variance. ns for a non-significant effect, ^∗^*P* < 0.05; ^∗∗^*P* < 0.01; ^∗∗∗^*P* < 0.001.*

**FIGURE 2:**
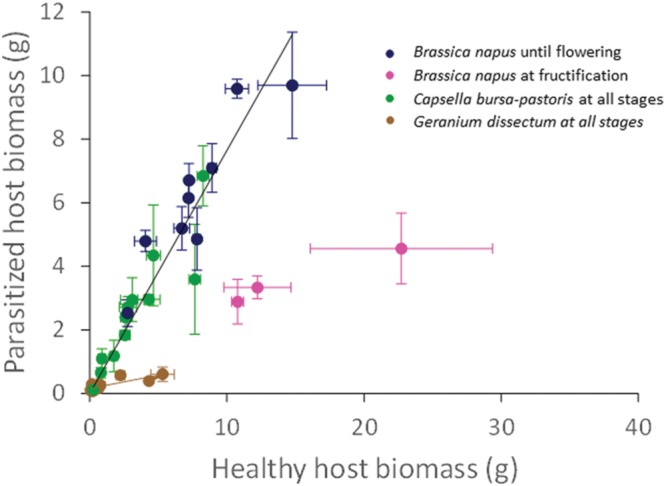
**Correlation between parasitized (P) and healthy (H) host biomass per host species.** Data are mean ± SE for three independent replicates (*n* = 3). The black line is for both *Capsella bursa-pastoris* at all stages and *Brassica napus* until flowering only (y = 0.82x; *R*^2^ = 0.97). The brown line is for both *Geranium dissectum* at all stages (y = 0.10x; *R*^2^ = 0.77).

The biomass of the parasitized host plants was positively and linearly related to that of the healthy host plants (*P* < 0.001). The regression parameters did not depend on light availability (*P* = 0.55) but varied with the host species (*P* < 0.001) and its phenological stage (*P* < 0.001). In *B. napus* (except at fructification) and *C. bursa-pastoris*, healthy and parasitized plant biomasses were correlated according to a single relationship (*P* < 0.001) that was valid whatever the host species (*P* = 0.15), the light level (*P* = 0.63) and the phenological stage (*P* = 0.09; **Figure 2**). The slope value of 0.82 g/g indicates that parasitism reduced host biomass by ca. 18% for *C. bursa-pastoris* whatever the phenological stage and *B. napus* from rosette to flowering stages. Parasitized plant biomass of *B. napus* at fructification stage was approximately 4 g, whatever light availability (*P* = 0.17). Biomasses of healthy and parasitized *G. dissectum* plants were also correlated (*P* < 0.001), with an average slope of 0.10 g/g showing that parasitism reduced host biomass by ca. 90% (average over all light levels) which is much higher than for the other host species. The regression slope varied with light level (*P* = 0.004) and host phenological stage (*P* = 0.007) but these effects were negligible (partial *R*^2^ = 0.09 and 0.10, respectively). A similar regression analysis was performed on the pathosystem biomass, i.e., the sum of both host and parasite biomass, giving similar conclusions (Supplementary Data Sheet 3).

To analyze the tolerance of host species to parasitism, biomass loss per host plant was expressed relatively to parasite biomass. Averaged over the light levels at host fructification, values were 1.9 ± 0.1 g/g for *B. napus*, 2.4 ± 1.3 g/g for *C. bursa-pastoris*, and 14.4 ± 8.8 g/g for *G. dissectum* showing that *G. dissectum* was much less tolerant to *P. ramosa* than the other two species.

### Host Biomass Distribution per Compartment

**FIGURE 3:**
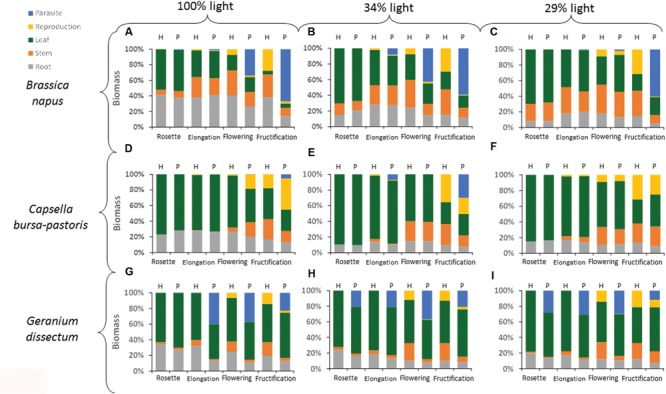
**Distribution of biomass per compartment for each host species × host phenological stage × light level combination.** H is for healthy plants and P for parasitized plants. Data are means for three independent replicates. The part-labels from **(A)** to **(I)** show the different host species per light level combinations.

The biomass distribution per compartment of the host plant (leaves, stems, roots, and reproductive organs) was calculated by dividing the biomass of a given compartment by the biomass of the pathosystem, including both host biomass and parasite biomass if any (**Figure 3**). Whatever the compartment, host species (*P* < 0.001), light level (*P* < 0.024), host phenological stage (*P* < 0.001), and parasitism (*P* < 0.004) all affected biomass distribution. Except for leaves, the effect of the parasitism also varied with host species (*P* < 0.001), phenological stage (*P* < 0.006), and light availability (*P* < 0.01). The effect of parasitism on leaf biomass distribution varied with host phenological stage (*P* = 0.002) but not with host species (*P* = 0.08) and light level (*P* = 0.17).

The biomass distribution per compartment was analyzed per host species (**Table 5**). For *B. napus*, the biomass distribution in leaves was affected by parasitism at the two lowest light levels. However, this effect was minor compared to that of the other factors (**Table 5**). For stem, root and reproductive compartments, the adverse effect of parasitism increased with the host phenological stages progression (**Figure 3**). The compartments could be ranked according to their sensitivity to parasitism. At *B. napus* fructification, the reproductive compartment was the most severely affected (averaged over all light levels, reproductive-biomass proportion was reduced by 97% for parasitized vs. healthy plants), followed by stems (reduced by 66%), roots (reduced by 50%) and leaves (not affected).

**Table 5:** Effects of parasitism by *Phelipanche ramosa*, light level, host stage, and interactions on biomass distribution per compartment for each host species.

Host species	Factors	Leaf	Stem	Root	Reproduction
*Brassica napus*	Light	0.15^∗∗∗^	0.11^∗∗∗^	0.55^∗∗∗^	ns
	Stage	0.80^∗∗∗^	0.30^∗∗∗^	0.14^∗∗∗^	0.41^∗∗∗^
	Parasitism	ns	0.22^∗∗∗^	0.03^∗∗^	0.19^∗∗∗^
	Parasitism:light	0.01^∗∗∗^	ns	ns	ns
	Parasitism:stage	ns	0.18^∗∗∗^	0.05^∗∗^	0.37^∗∗∗^
	Light:stage	0.01^∗∗^	0.07^∗∗∗^	0.05^∗^	ns
	Parasitism:light:stage	ns	0.04^∗^	ns	ns
*Capsella bursa-pastoris*	Light	0.01^∗∗^	0.04^∗∗∗^	0.55^∗∗∗^	ns
	Stage	0.87^∗∗∗^	0.79^∗∗∗^	0.14^∗∗∗^	0.79^∗∗∗^
	Parasitism	ns	ns	ns	ns
	Parasitism:light	0.02^∗∗^	ns	ns	0.04^∗∗∗^
	Parasitism:stage	ns	0.04^∗∗∗^	ns	ns
	Light:stage	0.03^∗∗^	0.04^∗∗∗^	0.09^∗∗∗^	0.02^∗^
	Parasitism:light:stage	ns	0.04^∗∗∗^	ns	0.06^∗∗∗^
*Geranium dissectum*	Light	0.05^∗^	0.02^∗∗^	0.26^∗∗∗^	0.03^∗^
	Stage	0.25^∗∗∗^	0.41^∗∗∗^	0.33^∗∗∗^	0.43^∗∗∗^
	Parasitism	0.05^∗∗^	0.25^∗∗∗^	0.18^∗∗∗^	0.18^∗∗∗^
	Parasitism:light	ns	ns	0.06^∗∗∗^	ns
	Parasitism:stage	0.19^∗∗∗^	0.17^∗∗∗^	ns	0.18^∗∗∗^
	Light:stage	0.09^∗^	0.04^∗∗^	ns	0.04^∗^
	Parasitism:light:stage	ns	0.03^∗^	0.03^∗^	ns

*Partial *R*^2^-values determined by analysis of variance. ns for a non-significant effect, ^∗^*P* < 0.05; ^∗∗^*P* < 0.01; ^∗∗∗^*P* < 0.001.*

Generally, in *C. bursa-pastoris*, parasitism did not affect the distribution of biomass, whatever the compartment (**Figure 3**; **Table 5**). Biomass allocation to leaves and reproductive compartment was though affected by parasitism at the highest light level whereas biomass allocation to stems was reduced at host fructification. However, these effects were minor compared to the direct effects of the other factors (low partial *R*^2^-values associated to the effects of interactions between factors in **Table 5**).

In *G. dissectum*, parasitism generally decreased biomass allocation to host compartments to the benefit of the parasite compartment (**Figure 3, Table 5**). The magnitude of the parasitism effect varied for all compartments (except roots) with host phenological stage (**Table 5**). At host fructification, the ranking of the host compartments according to their sensitivity to parasitism was identical to that for *B. napus*: the reproductive compartment was the most reduced (averaged over all light levels, reproductive-biomass proportion was reduced by 75% for parasitized vs. healthy plants), followed by stems (reduced by 66%), roots (reduced by 33%), and leaves (increased by 20%).

### *Phelipanche ramosa* Biomass

Host species, phenological stage and light level all affected absolute values of total *P. ramosa* biomass (including aboveground and belowground biomass) per host plant (**Figure 1**; **Table 6**). The main effects were host species and phenological stage. Parasite biomass was larger on *B. napus* than on the other two species. For *B. napus*, host phenological stage was the main factor affecting *P. ramosa* biomass per host plant and, for *C. bursa-pastoris*, it was the only significant factor (**Table 6**). For both species, *P. ramosa* biomass increased with host stage progression (**Figure 1**). For *G. dissectum*, light level had a greater impact (**Table 6**): depending on the light level, parasite biomass was the largest either at host elongation or flowering stage (**Figure 1**).

**Table 6:** Effects of light level, host stage and interaction on *Phelipanche ramosa* total biomass for each host species.

Host species	Factors	*F*-values
*Brassica napus*	Light level	0.10^∗∗^
	Host stage	0.62^∗∗∗^
	Interaction	0.12^∗^
*Capsella bursa-pastoris*	Light level	ns
	Host stage	0.26^∗∗^
	Interaction	ns
*Geranium dissectum*	Light level	0.33^∗∗∗^
	Host stage	0.22^∗∗∗^
	Interaction	0.29^∗∗∗^
All species	Host species	0.24^∗∗∗^
	Host stage	0.18^∗∗∗^
	Light level	0.03^∗∗∗^
	Species:stage	0.28^∗∗∗^
	Species:light	0.05^∗∗∗^
	Stage:light	0.03^∗^
	Species:stage:light	0.07^∗∗∗^

*Partial *R*^2^-values determined by analysis of variance. ns for a non-significant effect, ^∗^*P* < 0.05; ^∗∗^*P* < 0.01; ^∗∗∗^*P* < 0.001.*

**FIGURE 4:**
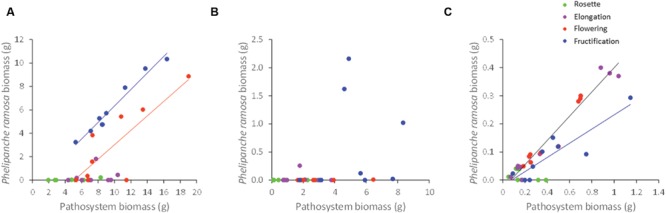
**Correlation between *Phelipanche ramosa* biomass and pathosystem biomass (including host and parasite biomasses) for the three host species, namely **(A)***Brassica napus*, **(B)***Capsella bursa-pastoris*, and **(C)***Geranium dissectum*.** Each point on the figure is a replicate. For *Brassica napus* the red and blue lines are for flowering stage (y = 0.62x-3.15; *R*^2^= 0.68) and fructification stage (y = 0.70x-0.70; *R*^2^= 0.96), respectively. For *Geranium dissectum*, the black line is for both elongation and flowering stages (y = 0.43x-0.03; *R*^2^= 0.95) and the blue line is for fructification stage (y = 0.25x-0.02; *R*^2^= 0.80).

Parasite biomass per host plant was positively and linearly related to the biomass of the pathosystem including both host and parasite biomasses (*P* < 0.001). Host phenological stage (*P* < 0.001), host species (*P* < 0.001), and light level (*P* < 0.001) all influenced the regression parameters (**Figure 4**). For *B. napus*, the parameters of the regression varied with host stage (*P* < 0.001) but not with the light level (*P* = 0.23) and the interaction between both factors (*P* = 0.17). Parasite biomass was significantly correlated to pathosystem biomass at host flowering (*P* = 0.006) and fructification (*P* < 0.001). The slopes of the correlations (0.62 and 0.70 g/g at flowering and fructification, respectively) did not differ between both stages (*P* = 0.65), showing that *P. ramosa* biomass increased in similar proportions (around 66%) with increasing pathosystem biomass (**Figure 4A**). Y-intercepts differed between host stages (*P* = 0.003) because of the larger parasite biomass at host fructification than at flowering for a given pathosystem biomass. The difference in y-intercept suggests that, at flowering stage, a larger minimum amount of pathosystem biomass is needed before any measurable parasite biomass is being produced.

For *G. dissectum*, as for *B. napus*, the regression parameters varied with the host stage (*P* < 0.001) but not with the light level (*P* = 0.96) and the interaction between both factors (*P* = 0.22). Parasite biomass was significantly correlated to that of the pathosystem at all host stages (*P* < 0.001), except rosette (*P* = 0.19). Slopes (*P* = 0.49) and y-intercepts (*P* = 0.99) were not different between elongation and flowering stages (slope value at 0.43 g/g) while the slope was lower (slope value at 0.25 g/g) and y-intercept was higher at fructification stage (*P* < 0.001; **Figure 4C**). The lower slope value at fructification indicated that the increase in *P. ramosa* biomass with increasing pathosystem biomass was less than at elongation and flowering.

For *C. bursa-pastoris* with its small number of attached *P. ramosa* plants (see Parasite Number and Phenology Across Host Species), most of the *P. ramosa* biomass values were close to nil, except at host fructification (**Figure 4B**). Parasite biomass was not correlated to pathosystem biomass, whatever the host phenological stage.

### Aboveground *P. ramosa* Shoot Number and Biomass

**FIGURE 5:**
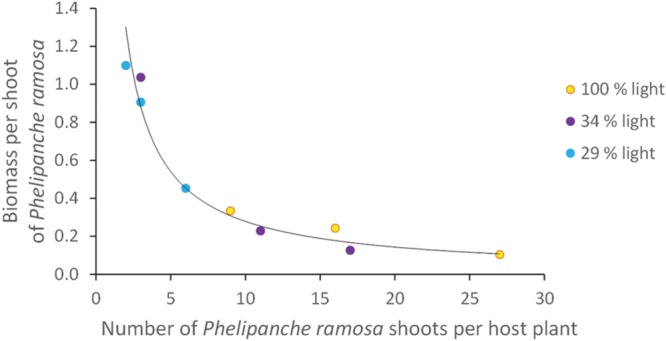
**Correlation between the biomass per shoot of *Phelipanche ramosa* and the number of *Phelipanche ramosa* shoots for *Brassica napus* at three different light levels.** Each point on the figure is a replicate. The line is a power function (y = 2.53x^-0.96^; *R*^2^ = 0.96).

The number of *P. ramosa* aboveground shoots per host plant was analyzed in relation to the aboveground biomass per shoot of *P. ramosa*. The analysis was performed for *B. napus* which was the only host species on which *P. ramosa* produced several aboveground shoots (see Parasite Number and Phenology Across Host Species). Light level did not significantly affect either the number of shoots per host plant (*P* = 0.12) nor the biomass per shoot (*P* = 0.20) nor the total aboveground parasitic biomass per host plant (*P* = 0.21) at 2.8 ± 0.5 g/g. Nonetheless, as the number of *P. ramosa* shoots increased, the biomass of each shoot decreased following a negative power relation (**Figure 5**), regardless of the light level.

## Discussion

### Response to *P. ramosa* Differed between Host Species

The effect of *P. ramosa* on host growth varied across host species, with host biomass losses ranging from 34 to 84% at host fructification. In the literature, the values of host biomass loss due to parasitism by broomrape species range from 15 to 72% (Dale and Press, 1998; Hibberd et al., 1998; Manschadi et al., 2001; Lins et al., 2007; Mauromicale et al., 2008). These values stem from independent studies differing on experimental design in terms of parasitic and host plant species, phenological stages at data collection, environmental conditions, and parasitic seed density. Even though our study considered only one parasitic plant species at a constant seed bank density and measurements were made at similar phenological stages for all host species, the range of values of biomass loss due to parasitism was as large as that found in literature.

*B. napus* and *C. bursa-pastoris* displayed similarities in their response to *P. ramosa*. Firstly, their tolerance to *P. ramosa*, i.e., their capacity to endure parasitism with minor losses of productivity, was similar. Secondly, the analysis of the correlation between the biomasses of parasitized and healthy hosts showed that the proportion of biomass loss due to parasitism was similar, except at host fructification when *B. napus* was more sensitive than *C. bursa-pastoris*. Thirdly, for both host species, the effect of parasitism on host biomass was maximal toward the end of the host life (fructification). Fourthly, the proportion of biomass loss due to parasitism was independent of the light level. These similarities between *B. napus* and *C. bursa-pastoris* could be due to their affiliation to the same botanical family. The lower resistance (characterized in our study by the number of parasite attachments) of *B. napus* compared to *C. bursa-pastoris* could be due to the *P. ramosa* pathovar that was used in this study, corresponding to a *B. napus* pathovar.

In contrast, *G. dissectum* was much more responsive to *P. ramosa*, with many more *P. ramosa* attachments reflecting a lower resistance of this host species. In spite of this, very few *P. ramosa* shoots emerged on this host. This could be due to (i) a genetic component of late-resistance in the host and/or (ii) an effect of high competition for host-derived nutritive resources between parasite individuals. Moreover, this species showed much more host biomass loss per g of parasite biomass, reflecting a lower tolerance to *P. ramosa*. Finally, for this host species, the maximal effect of parasitism occurred earlier in the host life-cycle (i.e., flowering). The dissimilarities between *G. dissectum* and the other two species could be linked to their different botanical families (Geraniaceae vs. Brassicaceae). Part of differences could though also be explained by the fact that this species was not grown at exactly the same period of time as the two other host species, though with identical parasite seed densities.

The relative timing of host and parasite stages seems to be a major factor for explaining differences in host tolerance among host species. The parasite was earlier on *G. dissectum* and consequently probably disrupted host plant functioning more. Parasite phenology was delayed on *C. bursa pastoris*, and therefore competition for assimilates tipped in favor of the host, with a lower impact on host growth. Considering a single host species, Manschadi et al. (1996) showed that the impact of the parasite *Orobanche crenata* on its host *Vicia faba* depended on the synchronization between parasite and host phenological stages. In our study, it is difficult to determine whether the relationship between host sensitivity and parasite precocity stem strictly from differences between plant species or also from differences in growth conditions (as *G. dissectum* was grown at a period of time different from that of the other two species). Nonetheless, both our results and Manschadi et al. (1996), suggest that this relationship could exist both at the intra- and inter-specific level.

### Host Reproductive Compartment was the Most Severely Affected by Parasitism

Broomrapes plants are known to compete strongly for resources within host plants, causing significant changes in biomass distribution (Barker et al., 1996; Hibberd et al., 1998; Lins et al., 2007). Here, for *B. napus* and *G. dissectum*, parasitism by *P. ramosa* strongly affected biomass partitioning among compartments, namely leaf, stem, root and reproductive compartments. For *C. bursa-pastoris*, no direct effect of parasitism was observed on biomass partitioning, probably because this host species was not very sensitive to *P. ramosa* (see Response to *P. ramosa* Differed between Host Species) and because the parasite produced little biomass on this host (see Host Biomass Loss Due to Parasitism is Not Systematically Invested in *P. ramosa* Biomass). For *B. napus* and *G. dissectum*, the most severely affected compartment was the reproductive compartment, particularly for *B. napus*. The stem compartment was the second most affected, followed by roots, whereas the leaf compartment was not reduced by parasitism whatever the host species. A similar ranking of the host compartments was identified for other broomrape species (Barker et al., 1996; Manschadi et al., 1996; Hibberd et al., 1998; Grenz et al., 2008). Broomrape species act as an additional sink that competes with host compartments for assimilates. The strongest impact of parasitic plants on host reproductive growth was shown by Manschadi et al. (2001, 2004). It could be related to the synchronism between the periods of host reproductive growth and parasite shoot growth. Conversely, the other host compartments (roots, stems, and leaves) are already well-established at the time when parasite shoots start to require large amounts of assimilates for their growth. Even if the proportion of leaves was little affected by parasitism, plant photosynthesis was probably reduced as suggested by the substantially lower total biomass production of the pathosystem compared to total biomass production of healthy plants for most of our experimental treatments. In literature, the effect of parasitism on host photosynthesis is pathosystem dependent, with studies showing that photosynthesis is unaffected (example of *Orobanche minor* in Dale and Press, 1998) whereas others report a reduced photosynthesis, in particular caused by *P. ramosa* (Mauromicale et al., 2008).

### Host Biomass Loss Due to Parasitism is not Systematically Invested in *P. ramosa* Biomass

Parasite sink strength was evaluated by the slope of the linear regression of parasite biomass vs. pathosystem biomass. It could not be evaluated for *C. bursa-pastoris* on which parasite biomass was negligible. On the two other species, *P. ramosa* was shown to adapt its sink strength to the host species and the host stage with, for a given host species and host stage, *P. ramosa* extracting a constant proportion of host assimilates whatever the host growth rate. To our knowledge, the present study is the first to analyze the effects of the host species and phenological stages on biomass allocation to the parasite. It allows ranking host species as a function of *P. ramosa* sink strength, with the lowest sink strength on *C. bursa-pastoris*, followed by *G. dissectum* and then *B. napus*. The highest sink strength on *B. napus* is consistent with the highest number of *P. ramosa* attachments and emerged shoots on this host species. The use of *P. ramosa* seeds from the *B. napus* pathovar is probably the explanation. The lowest sink strength on *C. bursa-pastoris* is consistent with the lowest sensitivity of this host species to *P. ramosa* (see Response to *P. ramosa* Differed between Host Species). However, the host species with the highest sensitivity to *P. ramosa* (*G. dissectum*) is not the species generating the highest *P. ramosa* sink strength (*B. napus*). In other words, *G. dissectum* was very sensitive to *P. ramosa* parasitism but *P. ramosa* was poorly efficient on this host, meaning that the host biomass loss due to parasitism was only poorly invested in *P. ramosa* biomass, contrary to what happened for *B. napus*.

### Rethinking the Host Concept

While parasite biomass was significant on the three species, the parasite did not reproduce, questioning the host status the studied species. A host is defined as a species which allows the parasite to attach and which confers benefits to the parasite in terms of growth and reproduction. However, it is established that a greater spectrum of responses are displayed by potential hosts (from true host to non-host and all stages in between) especially in the case of parasitic plants that are not host-specific, i.e., able to parasitize different host species, such as *P. ramosa* (Cameron et al., 2006). Our results show that, even though *P. ramosa* does not reproduce, it can generate large biomass losses for the plant on which it is attached. Thus, the three studied species cannot be considered as non-host. Moreover, *P. ramosa* was previously shown to reproduce on these species (Supplementary Data Sheet 1; Boulet et al., 2001; Gibot-Leclerc et al., 2012). Altogether, these findings indicate that variations in the host status are possible for a given species. As discussed above, variations in synchronization between parasite and host phenological stages, due to variations in environmental conditions, could explain why *P. ramosa* is not always able to reproduce on a given host species. An improper synchronization for the parasite could result in not enough time for the parasite to complete its biological cycle, and/or a too large nutrient flow extracted by the parasite, causing host death.

### Parasitic Aboveground Biomass per Shoot Decreased with Shoot Number per Host Plant

For *B. napus*, the aboveground biomass of individual *P. ramosa* shoots decreased with increasing shoot number per host plant, following a single relationship. Similar relationships were observed for two broomrape species, including *P. ramosa* (Hibberd et al., 1998; Mauromicale et al., 2008). Our study is the first to analyze the effect of host growth rate, modulated by the light level, on this relationship, showing that the relationship remained unchanged, whatever the host growth rate. Our data are consistent with Hibberd et al. (1998) in concluding that, as the number of parasite shoots increased, competition for assimilates between individual shoots decreased their individual biomass. Without such a regulation, i.e., if individual shoot biomass did not decrease with shoot number, a very high resource extraction by the parasite could potentially severely reduce host growth and consequently compromise parasite biomass and survival (Lins et al., 2007; Hautier et al., 2010).

### Agronomic Implications

This study provides clues on the effects of the trophic relationships between *P. ramosa* and the weed flora on parasite soil seed bank in cropping systems. It is established that each of the studied host species can induce *P. ramosa* reproduction (see Gibot-Leclerc et al., 2012 for *B. napus*; Boulet et al., 2001 for *C. bursa*-pastoris; Supplementary Data Sheet 1 for *G. dissectum*). Even though *P. ramosa* did not reproduce in our study, our findings allow comparing the three host species. Parasite biomass was much lower on the two weed species than on *B. napus*. As seed production is known to be correlated to plant biomass (e.g., Lutman et al., 2011), our results suggest that these two weed species would contribute little to parasite seed bank. They could possibly even contribute to reduce rather than increase *P. ramosa* seed bank. Indeed, even though more than 50 *P. ramosa* individuals were fixed per *G. dissectum* plant, only few of them emerged and none produced seeds. This could be particularly true during summer fallow where the sheer abundance of weeds could compensate their small root system (and thus the resulting lower stimulation of fatal parasite germination) without hindering cash crop development. In the future, conducting new studies on a range of potential host weed species will be helpful to discriminate the weed species promoting from those impeding *P. ramosa* dissemination particularly checking parasite seed production ability. In order to characterize possible variations in the synchronization between host and parasite phenology for a given host species, these studies will have to consider different cohorts for the weed species which are able to germinate at different periods during the year (e.g., *C. bursa-pastoris* and *G. dissectum*; Mamarot and Rodriguez, 2014). Such studies should help to identify the host weed species that should be controlled in priority in order to manage *P. ramosa* seed bank in cropping systems.

Parasitism reduced host growth less in *C. bursa-pastoris* than in *G. dissectum*, suggesting that *P. ramosa* can affect plant species differently and therefore could modify weed community assembly in cropping systems. This result is supported by studies at the community level on other parasitic plants: some species (i.e., highly sensitive host species) were shown to be penalized by the presence of the parasite, leading to a lower resources uptake to the benefit of other species in the community (Gibson and Watkinson, 1991; Ameloot et al., 2005).

Our study tested only three host species. Considering a wider range of species, especially weed species, would be necessary to go further in the analysis of a host species effect. Our next step will be to integrate our data into a mechanistic model of the effects of cropping systems on parasite population dynamics. While previous models integrated interactions with the host crop species only (Grenz et al., 2006), a model that integrates parasite population dynamics in interaction with non-parasitic weed hosts will be considered (Colbach et al., 2011). Mechanistic models are particularly useful to predict and understand complex systems (Rossing et al., 1997; Colbach et al., 2014). They will thus help to further analyze the effect of parasitic plant species on weed growth and community assembly and to design cropping systems for controlling *P. ramosa*.

## Conclusion

The intensity of the response to *P. ramosa* differed among host species and, depending on the host species, host biomass loss due to parasitism was not systematically invested in *P. ramosa* biomass. The parasite adapted its growth to host biomass production and the proportion of pathosystem biomass allocated to the parasite generally increased with host stage progression. Results suggests that some host weed species could contribute to increase while others could contribute to reduce *P. ramosa* soil seed bank. If confirmed, weed management may have to be rethought in order to restrain *P. ramosa* dissemination in infested fields and in fields with risks of infestations.

## Author Contributions

DM, SG-L, and NC designed the study. AG, CR, and FS collected the data. DM, AG, OP, MF-A, and NC analyzed and interpreted the data. All the authors contributed to manuscript writing.

## Conflict of Interest Statement

The authors declare that the research was conducted in the absence of any commercial or financial relationships that could be construed as a potential conflict of interest.

The reviewer AVA and handling Editor declared their shared affiliation, and the handling Editor states that the process nevertheless met the standards of a fair and objective review.

## Footnotes

1 We initially aimed at 60 and 30% light levels for the two shaded treatments but the continuous light measurements during the experiment showed the shading to be more similar than planned, despite preliminary tests.
