# Supplementary material for: Trophic Relationships between the Parasitic Plant Species Phelipanche ramosa (L.) and Different Hosts Depending on Host Phenological Stage and Host Growth Rate
Source: Front Plant Sci. 2016 Jul 13;7:1033. doi: 10.3389/fpls.2016.01033 (PMC4942479; doi:10.3389/fpls.2016.01033)

***Supplementary material***

**Trophic relationships between the parasitic plant species *Phelipanche ramosa* (L.) Pomel and crop and weed host species**

Delphine Moreau^*^, Stéphanie Gibot-Leclerc, Annette Girardin, Olivia Pointurier, Carole Reibel, Florence Strbik, Mónica Fernández-Aparicio, Nathalie Colbach

*** Correspondence:** Corresponding Author: [delphine.moreau@dijon.inra.fr](mailto:delphine.moreau@dijon.inra.fr)

**Supplementary Data sheet 1.** Fructified *Phelipanche ramosa* attached to *Geranium dissectum* in a severely infested *Brassica napus* field located in Saint-Jean-d'Angély (Charente-Maritime, France) in July 2001.


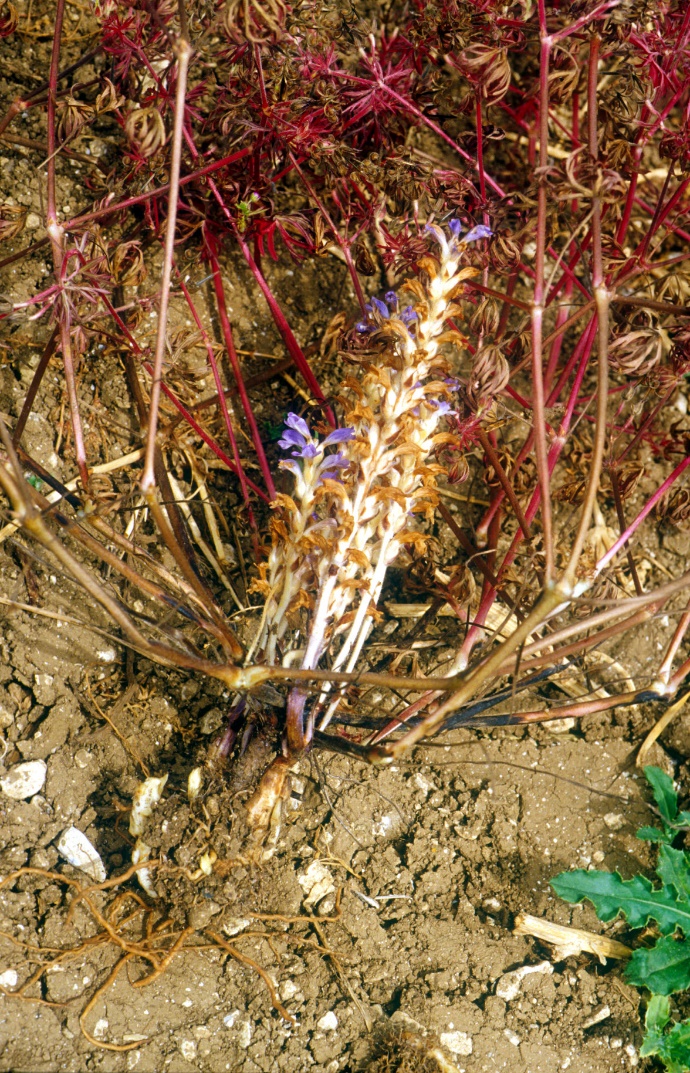

Supplement: Supplementary file 1 [file Data_Sheet_1.DOCX]
