# Supplementary material for: Trophic Relationships between the Parasitic Plant Species Phelipanche ramosa (L.) and Different Hosts Depending on Host Phenological Stage and Host Growth Rate
Source: Front Plant Sci. 2016 Jul 13;7:1033. doi: 10.3389/fpls.2016.01033 (PMC4942479; doi:10.3389/fpls.2016.01033)
Supplement: Supplementary file 2 [file Data_Sheet_2.DOCX]

***Supplementary material***

**Trophic relationships between the parasitic plant species *Phelipanche ramosa* (L.) Pomel and crop and weed host species**

Delphine Moreau^*^, Stéphanie Gibot-Leclerc, Annette Girardin, Olivia Pointurier, Carole Reibel, Florence Strbik, Mónica Fernández-Aparicio, Nathalie Colbach

*** Correspondence:** Corresponding Author: [delphine.moreau@dijon.inra.fr](mailto:delphine.moreau@dijon.inra.fr)

**Supplementary Data sheet 2**: Harvest dates for the three host species, the four phenological stages and the three light levels. Dates are given in in days and degree-days after sowing. Degree-days are calculated according to Bonhomme (2000) with a base temperature at 0°C for *B. napus* (Habekotté, 1993) and *G. dissectum* (Guillemin et al., 2013) and 4°C for *C. Bursa-pastoris* (Guillemin et al., 2013). As parasitism did not delay host phenology in our study, parasitized and healthy plants were harvested at the same date for a given host species in a given light treatment.

| Host species | Light level | Rosette | | Elongation | | Flowering | | Fructification | |
| --- | --- | --- | --- | --- | --- | --- | --- | --- | --- |
|  |  | Days | Degree-days | Days | Degree-days | Days | Degree-days | Days | Degree-days |
| *Brassica napus* | 100% | 125 | 1604 | 182 | 2019 | 200 | 2212 | 249 | 2938 |
|  | 34% | 126 | 1610 | 186 | 2064 | 207 | 2314 | 250 | 2962 |
|  | 29% | 127 | 1616 | 194 | 2143 | 208 | 2328 | 251 | 2990 |
| *Capsella bursa-pastoris* | 100% | 123 | 1100 | 130 | 1115 | 159 | 1180 | 196 | 1375 |
|  | 34% | 124 | 1103 | 165 | 1205 | 189 | 1347 | 252 | 2010 |
|  | 29% | 124 | 1103 | 180 | 1275 | 193 | 1364 | 256 | 2100 |
| *Geranium dissectum* | 100% | 62 | 978 | 112 | 1691 | 138 | 2176 | 168 | 2787 |
|  | 34% | 76 | 1167 | 110 | 1655 | 151 | 2442 | 169 | 2803 |
|  | 29% | 77 | 1182 | 111 | 1673 | 152 | 2464 | 173 | 2881 |

Bonhomme, R. (2000). Bases and limits to using ‘degree.day’ units. *European Journal of Agronomy* 13, 1-10. doi:10.1016/S1161-0301(00)00058-7

Guillemin, J.P., Gardarin, A., Granger, S., Reibel, C., Munier-Jolain, N., and Colbach, N. (2013). Assessing potential germination period of weeds with base temperatures and base water potentials. *Weed Research* 53, 76-87. doi: 10.1111/wre.12000

Habbekotté, B. (1993). Quantitative analysis of pod formation, seed set and seed filling in winter oilseed rape (*Brassica napus* L.) under field conditions. *Field Crops Research* 35, 21-33.
