# Supplementary material for: Trophic Relationships between the Parasitic Plant Species Phelipanche ramosa (L.) and Different Hosts Depending on Host Phenological Stage and Host Growth Rate
Source: Front Plant Sci. 2016 Jul 13;7:1033. doi: 10.3389/fpls.2016.01033 (PMC4942479; doi:10.3389/fpls.2016.01033)
Supplement: Supplementary file 3 [file Data_Sheet_3.DOCX]

***Supplementary material***

**Trophic relationships between the parasitic plant species *Phelipanche ramosa* (L.) Pomel and crop and weed host species**

Delphine Moreau^*^, Stéphanie Gibot-Leclerc, Annette Girardin, Olivia Pointurier, Carole Reibel, Florence Strbik, Mónica Fernández-Aparicio, Nathalie Colbach

*** Correspondence:** Corresponding Author: [delphine.moreau@dijon.inra.fr](mailto:delphine.moreau@dijon.inra.fr)

**Supplementary Data sheet 3.** Correlation between the parasitized host plant biomass and the pathosystem biomass

The biomass of the pathosystem (i.e. the sum of both host and parasite biomass) was positively and linearly related to the biomass of healthy plants (P<0.001; see Figure below). The regression parameters depended on host species (P<0.001), phenological stage (P<0.001) and light availability (P=0.009).

Considering both *B. napus* and *C. bursa-pastoris*, regressions parameters did not differ between species (P=0.10) and phenological stages from rosette to flowering (P=0.48), with a single relationship (P<0.001; Figure B1). The slope value of 0.92 g/g indicates that parasitism reduced host biomass by ca. 8%. Regression parameters differed between light levels but the associated partial R² was negligible (R²<0.01). At fructification stage, regressions parameters differed between species (P=0.008) but not with the light level (P=0.17).

For *G. dissectum*, the biomass of the parasitized host plants was positively and linearly related to the biomass of the pathosystem (P<0.001), with an average slope of 0.22 g/g which is much lower than for the other host species. The regression slope varied with light level (P=0.003; partial R² values=0.22) and host phenological stage (P=0.004; partial R² values=0.08).

**Correlation between the pathosystem biomass (the sum of host and parasite biomass) for parasitized plant and the biomass healthy plants.** Data are means +/- SE for three independent replicates (n=3). The black solid line is for *Capsella bursa-pastoris* and *Brassica napus* until flowering only for both species (y=0.92x; R²=0.98). The brown line is for both *Geranium dissectum* at all stages (y=0.22x; R²=0.66). The dotted line is the bisector line.
